# Multiscale determinants of Pacific chorus frog occurrence in a developed landscape

**DOI:** 10.1007/s11252-020-01057-4

**Published:** 2020-10-08

**Authors:** Jemma Green, Purnima Govindarajulu, Eric Higgs

**Affiliations:** 1 School of Environmental Studies, University of Victoria, 3800 Finnerty Rd, Victoria, British Columbia V8P 5C2 Canada; 2 BC Ministry of Environment and Climate Change Strategy, 525 Superior Street, Victoria, British Columbia V8V 0C5 Canada

**Keywords:** *Pseudacris regilla*, Amphibian, Urban ecosystem, Constructed wetlands, Ecological design, Occupancy model

## Abstract

Pacific chorus frog (*Pseudacris regilla*) populations have persisted despite urban and rural development throughout the species’ range; yet it is possible that *P. regilla*, like other anurans with which it historically co-occurred, will become extirpated from cities and suburbs if urbanization intensifies as predicted. An improved understanding of the conditions that enable this species to persist in developed landscapes is needed to identify and conserve suitable habitats. We investigated species-habitat relationships for *P. regilla* in a mixed urban-rural landscape in southwestern British Columbia, Canada, to identify potential criteria for habitat suitability. We conducted repeat auditory surveys of chorusing males at 52 potential breeding wetlands and modeled occupancy at 26 of these sites using local and landscape variables representing competing hypotheses and spatial scales of influence. The models that best explained *P. regilla* occupancy included a combination of terrestrial habitat and connectivity factors and the presence of non-native predators. We found that the proportion of impervious cover within 250 m of a wetland had the strongest negative impact on occupancy. Our findings suggest that availability of terrestrial habitat adjacent to breeding sites is the primary driver of species presence in the developed landscape. Conservation efforts should seek to limit impervious cover to less than 20% within a 250-m buffer around breeding wetlands. Further, restored and created wetlands in urban and rural areas may be more likely to support *P. regilla* if they are designed with a seasonal hydroperiod that excludes non-native aquatic predators and are placed in an area of high pond density.

**Electronic supplementary material:**

The online version of this article (10.1007/s11252-020-01057-4) contains supplementary material, which is available to authorized users.

## Introduction

Amphibians are experiencing dramatic population declines worldwide (Houlahan et al. 2000; Stuart et al. 2004; Hof et al. 2011), with an estimated 41% of species listed as threatened by the International Union for Conservation of Nature and Natural Resources (IUCN 2020). In North America, habitat loss and fragmentation by urban and rural development are among the leading causes of amphibian decline (Lehtinen et al. 1999; Baldwin and DeMaynadier 2009; Scheffers and Paszkowski 2012; Grant et al. 2016). Aquatic-breeding amphibians are particularly vulnerable to the impacts of development due to their requirement for multiple habitats to support their complex life history (Baldwin and DeMaynadier 2009), their relatively low vagility (Cushman 2006; Hillman et al. 2014), and their heightened sensitivity to environmental contaminants and stressors due to their unique physiology (Egea-Serrano et al. 2012). Nevertheless, recent research has encouraged cautious optimism about the oft overlooked conservation potential of urban ecosystems for amphibians. Some species have shown remarkable behavioural plasticity in habitat selection and resilience to landscape change (e.g., Brand and Snodgrass 2010; Saarikivi et al. 2013; Holzer and Lawler 2015). Furthermore, it is possible for urban and rural wetlands to support abundant amphibian populations (e.g. Riley et al. 2005), high species richness (e.g. Holzer 2014; Boissinot et al. 2019), and genetic diversity similar to habitats outside of developed areas (e.g. Garcia-Gonzalez and Garcia-Vazquez 2012).

Successful conservation of a species in urbanizing regions requires knowledge of their current distribution and the factors that contribute to habitat suitability. Yet knowledge of how distribution, abundance, and persistence is influenced by local and landscape processes is incomplete for many aquatic-breeding amphibians, even in the context of relatively undisturbed ecosystems (Semlitsch 2002; Nori et al. 2018). Still less is known about how these processes change with spatial scale and with the intensity of urbanization (Hamer and McDonnell 2008; Scheffers and Paszkowski 2012). This knowledge gap can be addressed, in part, with species-specific, multi-scale studies (Cushman 2006). Such studies can identify whether species occurrence at breeding sites is driven most strongly by local characteristics of the aquatic habitat, within which breeding and juvenile development occurs, or by characteristics of terrestrial habitat assessed at larger scales on the surrounding landscape, in which foraging, hibernation/estivation, and migration and dispersal movement occurs. By relating occurrence to habitat variables measured at multiple spatial scales, it may be possible to pinpoint the resolution, or scale of effect (Miguet et al. 2016), at which each habitat variable should be managed.

The Pacific chorus frog (*Pseudacris regilla*) is an apparent urban-adapted species for which habitat suitability criteria are poorly understood. *P. regilla* is found from British Columbia to Baja California (Matsuda et al. 2006). Unlike several aquatic-breeding amphibians with which it co-occurs, *P. regilla* populations have persisted despite urban and agricultural development throughout its range (Rorabaugh and Lannoo 2005). *P. regilla* is known to tolerate a relatively wide range of environmental stressors (e.g. Ovaska et al. 1997; Marco et al. 1999; Sparling and Fellers 2009) and has been observed in developed landscapes from Los Angeles, California to Greater Victoria, British Columbia (Riley et al. 2005; Holzer 2014). A better understanding of the habitat characteristics influencing *P. regilla* occupancy of aquatic and terrestrial environments in these landscapes is necessary to guide management of this species in the face of intensifying urban development.

With successful management, *P. regilla* could be an ideal flagship species to advance biodiversity conservation and habitat restoration initiatives in urban and rural areas of western North America. It is a non-threatening, readily-observed, and charismatic animal with positive associations: *P. regilla* choruses signal the arrival of spring and contribute to popular culture, providing the ambient noise for Hollywood’s night-time scenes. As an aquatic-breeding amphibian, *P. regilla* is an important component of the food web, both as predator and as prey, contributing to the flow of nutrients between aquatic and terrestrial environments and helping to control insect populations (Wells 2010; Bishop et al. 2014). Like other amphibians, *P. regilla* is a good indicator of ecosystem health due to its vulnerability to environmental stressors and pollutants and its complex habitat requirements (U.S. EPA 2002; Blaustein et al. 2003; but see Kerby et al. 2010). The presence of *P. regilla* is also a good indicator of connectivity between aquatic and terrestrial habitats for other species of equal or greater vagility, such as other amphibians, turtles, and small mammals, as it is unlikely to survive its annual migrations between these habitats without a movement corridor offering sufficient shelter and presenting few physical barriers.

In this study, we build on the body of knowledge of *P. regilla* ecology in the developed landscape by investigating the local and landscape-scale factors that drive breeding habitat occupancy in a mixed urban-rural landscape. Our objectives were to determine whether occupancy is most strongly influenced by (1) aquatic habitat quality, (2) the presence of non-native aquatic predators, (3) terrestrial habitat availability, (4) habitat connectivity, or (5) a combination of these local and landscape factors (Table 1), and to determine the scale of greatest influence of landscape factors. We modeled our hypotheses using occupancy analysis, predicting that *P. regilla* occupancy would be best explained by a combination of local and landscape variables reflecting the competing importance of multiple life history processes, rather than by variables linked to a single process or scale. A secondary objective was to compare *P. regilla* presence between urban and rural, and natural and constructed, wetlands. We predicted that *P. regilla* would not discriminate between urban and rural or natural and constructed wetlands, provided certain local and landscape criteria were met. Based on the relative explanatory power and scale of greatest influence of local and landscape-scale habitat variables in supported occupancy models, we propose habitat suitability criteria to guide conservation, restoration, and urban ecological design in the region.

**Table 1:** Summary of a priori hypotheses used to structure models of *Pseudacris regilla* occupancy, including covariates for occupancy probability and the scale(s) of analysis for each covariate

Hypothesis & global occupancy model structure	Covariates	Scale(s) of analysis
Wetland occupancy is driven by aquatic habitat quality *ψ*(DEPTH + AQVEG + CANOPY)	Water depth (+) Aquatic vegetation cover (+) Canopy cover (−)	Local
Wetland occupancy is driven by the presence of non-native aquatic predators *ψ*(BULLFROG + FISH)	American bullfrog P/A (−) Fish P/A (−)	Local
Wetland occupancy is driven by terrestrial habitat availability *ψ*(IMP) *ψ*(TREE)	Impervious cover (−) Tree cover (+)	Landscape: 50, 100, 150, 250, 500, 1000, 1500, 2000 m from pond
Wetland occupancy is driven by habitat connectivity *ψ*(PONDS + RD + WET + NEAR)	Number of ponds (+) Road density (−) Wetland cover (+)	Landscape: 50, 100, 150, 250, 500, 1000, 1500, 2000 m from pond
	Distance to nearest pond (−)	Landscape; constant across scales

Covariates are predicted as having a positive (+) or negative (−) influence on occupancy. Psi (*ψ*) is probability of occupancy

## Methods

### Study area

This study was conducted in Saanich, British Columbia, a 104 km^2^ municipality in the greater Victoria region of southern Vancouver Island, Canada. Historically, Douglas-fir (*Pseudotsuga menziesii*) forest was the dominant land cover, interspersed with open prairie and rich in wetlands (Bjorkman and Vellend 2010), including marshes, vernal pools, streams, lakes, and wet meadows that presumably supported abundant amphibians. Since European settlement, over 75% of wetlands and 95% of open prairie in the region have been lost to agricultural and urban development (GOERT 2003; Cox and Cullington 2009). Saanich is now situated within the second-most populous metropolitan area in the Province of British Columbia. It is divided approximately equally into Rural Saanich and Urban Containment, a zoning arrangement that has tempered ongoing and intensifying development pressures. Nevertheless, throughout Saanich, remnant wetlands, as well as constructed ponds and ditches, continue to be affected directly or indirectly by urban and rural development.

### Site selection

We used a stratified random sampling design to address questions of whether species occurrence and habitat relationships differ between urban and rural landscapes, between lentic and lotic wetlands, and between natural and constructed wetlands. A detailed map of all freshwater features (hereafter referred to as wetlands) was created in ArcMap (version 10.5; ESRI 2017). Each mapped wetland was assigned the attributes of urban or rural, lentic or lotic, and natural or constructed (e.g., rural lentic constructed wetland, urban lotic natural wetland, etc.). We then used NOAA’s Sampling Design Tool for ArcGIS (NOAA 2013) to generate an equal number of randomly selected wetlands (study sites) within each strata, keeping a minimum distance of 500 m between each site to ensure spatial independence (Petranka et al. 2004; Grand et al. 2017).

**Fig. 1:**
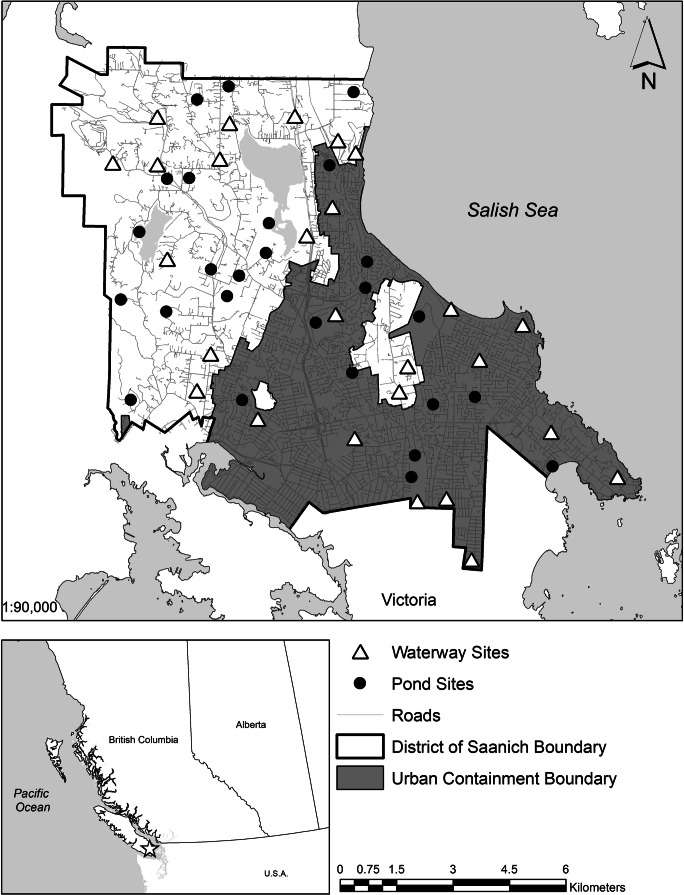
Map of surveyed sites in Saanich, British Columbia. Inset: Map of western Canada; the star indicates the location of the study area

Randomly selected wetlands were vetted in ArcGIS using a 2015 aerial orthophoto to confirm their presence. Several sites had access restrictions and were replaced by other randomly selected sites within that strata for which access was granted by the private landowner or public park agency. We then confirmed each site’s suitability with a site visit. A minimum hydroperiod of standing water until July, when the majority of *P. regilla* have metamorphosed, was used as the suitability criterion for ponds. The criteria for stream and ditch suitability included a minimum length of 100 m, still water or low flow, water depth exceeding 10 cm, and a hydroperiod extending until at least July. If a stream/ditch (hereafter collectively referred to as waterways) did not have standing water at the time of the site visit, a minimum channel depth of 0.5 m and the presence of hydrophytic vegetation (e.g., *Lysichiton americanus*, *Carex* spp., *Typha* spp.; Cox and Cullington 2009) were used as indicators of suitability. Sites that did not meet these suitability criteria were replaced by other randomly selected sites within that strata. A total of 52 wetlands were selected for study, including 12 urban ponds, 12 urban waterways, 14 rural ponds, and 14 rural waterways (Fig. 1).

### Data collection

#### Species presence/non-detection

We used call surveys to indicate species presence at a potential breeding wetland. Call surveys were conducted from mid-March to mid-May 2017, timed to coincide with the peak breeding season of *P. regilla* in this region*.* Surveys were conducted by a single observer between 30 min after sunset and 0100 h, which approximates the peak calling period of North American anurans (Weir and Mossman 2005). A survey consisted of 5 min of listening from a randomly selected point along the water’s edge (Dorcas et al. 2010). Surveys were repeated three times at each site to establish a detection history sufficient to estimate probability of detection (MacKenzie and Royle 2005). A site was considered occupied by *P. regilla* if one or more individuals were observed or heard calling.

#### Variables with potential impacts on detectability

During each survey, we collected data for variables that could impact *P. regilla* vocalization behaviour or the surveyor’s hearing ability, to use in modeling detection probability (MacKenzie et al. 2002, 2006). These variables included time since sunset, air and water temperature, wind speed, and ordinal codes representing precipitation intensity, moon brightness, and level of ambient noise disturbance (Weir and Mossman 2005) (Online Resource 1). If American bullfrogs—a non-native predator and competitor—were heard or observed, this was also noted. In accordance with the North American Amphibian Monitoring Program protocol, surveys were not conducted in winds of 20 km/h or higher, if precipitation was heavy enough to impact hearing ability, or if air temperature dropped below 5.6 °C (Weir and Mossman 2005). Relative humidity at the time of each survey and total daily rainfall were also considered. These measurements were collected from the nearest weather station to each site.

#### Local habitat characteristics

The variables measured at each wetland were chosen through a literature review and consideration of *P. regilla* life history characteristics. The covariates that we assessed for habitat quality were aquatic vegetation cover (Goldberg and Waits 2009; Holzer 2014; Hossack 2017), littoral water depth (Guderyahn et al. 2016), and canopy cover (Goldberg and Waits 2009) (Table 1). Hydroperiod may also be an important habitat factor for *P. regilla* (Guderyahn et al. 2016); however, we could not consider it as a covariate because the proportion of seasonal wetlands in our sample—a product of the scarcity of seasonal wetlands in the study area—was too low to permit analysis.

Habitat quality variables for 18 of 52 sites were measured opportunistically in late January 2017 (outside of *P. regilla*’s breeding season) as part of another study. Habitat quality variables for the remaining sites were measured between late March and mid-May 2017, to coincide with the breeding season. Aquatic vegetation cover, littoral water depth, and canopy cover were measured at multiple points at each site and then averaged. For ponds, these data were collected at the approximate cardinal direction points using a 1 m^2^ quadrat frame spanning 0–1 m distance from the shore; for waterway transects, data were collected from within the 1 m^2^ quadrat frame placed mid-channel at 0 m, 50 m, and 100 m points along the 100-m transect. Percent cover of aquatic vegetation was visually estimated by the same observer. Water depth was measured within each quadrat using a metre stick at a distance of 1 m from the water’s edge. We measured percent canopy cover over each quadrat using a densiometer.

In the study area, non-native aquatic predators of *P. regilla* include American bullfrogs (*Lithobates catesbeianus*), pumpkinseed sunfish (*Lepomis gibbosus*), goldfish (*Carassius auratus*), and smallmouth bass (*Micropterus dolomieu*). One-time surveys of non-native predator presence/non-detection were conducted at permanent pond sites in June and July 2017 using minnow traps (Shaffer et al. 1994; Skelly and Richardson 2010). Minnow traps were not deployed at sites where non-native fish and American bullfrogs were readily observed or in waterways, which are not known to support populations of these non-native predators.

#### Landscape-scale habitat characteristics

A literature review identified two relevant landscape-scale variables used to describe *P. regilla* terrestrial habitat: tree cover (Pearl et al. 2005; Goldberg and Waits 2009; Holzer 2014) and impervious surface cover, which includes buildings, roads, parking lots, and other built features of the urban environment (Rowe and Garcia 2014; Guderyahn et al. 2016). We identified four additional landscape-scale variables with potential to influence *P. regilla* occupancy based on studies of similar species and probable metapopulation dynamics: percent wetland cover (Johnson et al. 2013), road density (Marsh et al. 2017), number of ponds (Watts et al. 2015), and distance to the nearest pond (Marsh and Trenham 2001). These variables were grouped according to separate hypotheses of terrestrial habitat availability and connectivity (Table 1).

The scale of influence of a habitat variable can be defined as the spatial scale at which the strength of the relationship between species response (e.g. occurrence) and the amount of that variable (e.g. wetland cover) is the strongest (Quesnelle et al. 2015). The spatial scale at which landscape-scale habitat variables influence wetland occupancy for *P. regilla* is not well understood; therefore, we measured landscape variables within eight nested buffers of 50, 100, 150, 250, 500, 1000, 1500, and 2000 m around each wetland using ArcMap (version 10.5; ESRI 2017) to compare scale of influence during analysis.

Tree cover and impervious surface cover data were summarized within 1-ha grid cells using a 2015 aerial orthophoto provided by the Capital Regional District (Urban Forest Stewardship Initiative 2008). Tree cover refers to urban forests, which in this case includes relict forest and woodland patches of all stages of maturity, orchards, and ornamental plantings, to the scale of individual trees. We used a detailed waterbodies shapefile supplemented with a 2015 aerial orthophoto, provided by the District of Saanich, to map all wetland boundaries to a minimum area of 2 m^2^. We then calculated the distance to the nearest neighbour pond, percent wetland cover, and number of ponds at each scale using ArcMap’s Analysis Tools. We derived road density, calculated as summed length of all roads divided by the buffer area (m/m^2^; Simon et al. 2009), for each scale from the Digital Road Atlas layer published by the B.C. Ministry of Forests, Lands, Natural Resource Operations and Rural Development. Roads included all single and multi-lane roads and highways current to 2017.

### Statistical analysis

We used *P. regilla* call data to develop single-season occupancy models structured according to four a priori hypotheses (Table 1). Single season models incorporate two parameters: the probability of occupancy (*ψ*) and probability of detection (*p*) (MacKenzie et al. 2006). Occupancy analysis was conducted in the R software environment (R Core Team 2017) using the package ‘unmarked’ (Fiske and Chandler 2011). At each stage of the analysis, we used an information-theoretic approach to model selection, ranking models based on AICc, a second-order variant of Akaike’s information criterion that corrects for small sample size (n/k < 40) (Akaike 1973; Hurvich and Tsai 1989; Burnham and Anderson 2002).

We developed a global occupancy model for each hypothesis after completing four preliminary steps: (1) identifying and addressing non-linear and colinear relationships (Zuur et al. 2010); (2) scaling all continuous variables prior to modeling to standardize values across the many different scales of measurement (MacKenzie et al. 2006); (3) modeling detection probability; and (4) determining the scale of greatest impact for each landscape variable by comparing occupancy models of each variable at each scale.

Detection probability accounts for the sampling bias inherent in call surveys when the target species may go undetected even if present (MacKenzie et al. 2002, 2006). Variables with the potential to impact detection of *P. regilla* calls include sampling-specific variables and pond area, bullfrog presence, and fish presence. Pond area was included as a detection variable because the ability to detect calls may diminish with distance from the calling individuals. Bullfrogs and fish were included because there is some indication that *P. regilla* vocalization may be inhibited in close proximity to predators (Brattstrom and Warren 1955). To determine the best-fitting model for detection probability, we modeled detection variables individually and in all combinations of two covariates while holding occupancy probability constant. The maximum number of covariates in any given detection probability model was limited to two to avoid overfitting given the small sample size. The detection covariates from the most supported model (ΔAICc = 0) were then incorporated into subsequent occupancy models.

We inferred the scale of influence for scale-dependent landscape variables by comparing a set of eight occupancy models for each variable. Within each set, models included the detection covariates and a single landscape variable that varied in value according to the eight scales of measurement. The scale that appeared in the model with the most support (ΔAICc = 0) was used to select the covariate value for use in the global model for the respective hypothesis (Table 1).

For each hypothesis, the global occupancy model was ranked against more parsimonious subset models using AICc. Models with ΔAICc <2 from the top ranked model were considered to have similar support from the data (Burnham and Anderson 2002). The top models for each hypothesis were then compared to each other to determine the best model for *P. regilla* wetland occupancy in the developed (urban and rural) landscape. Akaike weights and effect sizes for *ψ* and *p* were compared to assess the relative explanatory power of each competing model. Finally, we tested the goodness of fit of the global model from which the top model was derived using a parametric bootstrap goodness of fit test (Burnham and Anderson 2002; MacKenzie and Bailey 2004). We used 5000 runs to test goodness of fit and estimate overdispersion (MacKenzie and Bailey 2004).

#### A posteriori modeling: combined model and relative variable importance

Previous studies of hylid frogs have found that models that included covariates from multiple spatial scales best explained wetland occupancy (e.g., Altmoos and Henle 2010; Fischer et al. 2015; Johnson et al. 2016). Therefore, after examining the results of the hypothesis model sets for occupancy, we created a multiscale model that combined variables from the most supported model for each hypothesis. This multiscale model was then ranked against the other models to determine if a model reflecting the competing importance of multiple life history processes better reflected the data. Finally, relative AICc weights of variables were used to determine the relative importance of each variable in explaining *P. regilla* occupancy.

## Results

Call surveys detected *P. regilla* at 18 of 26 ponds but only 1 of 26 waterway transects (a rural stream that had, at the transect location, temporarily flooded a field and developed the characteristics of a lentic habitat). Due to the small number of occurrences in waterways, these sites were excluded from further analysis and occupancy modeling was performed on the reduced set of 26 pond sites. *P. regilla* were detected at 42% of urban ponds and 93% of rural ponds. An equal number of natural and constructed ponds were occupied. At sites where *P. regilla* was detected, calls were heard an average of 2.3 times over three repeat surveys (median 2.5, range 1–3).

American bullfrogs were detected in 67% of occupied ponds and non-native fish predators were detected in 33% of occupied ponds. On average, occupied ponds had deeper littoral zones, greater aquatic vegetation cover, and less canopy cover than ponds with no detections (Table 2). Waterway sites were significantly shallower (U = 144, *p* = 2.0e-4) and shadier (U = 450, *p* = 0.02) than pond sites, as determined by a Mann-Whitney U test (*p* < 0.05).

**Table 2:** Descriptive statistics (range (mean)) for local habitat variables measured in ponds occupied and not occupied (no detections) by *Pseudacris regilla*, and in all waterway transects sampled

Local habitat variable	Ponds occupied (*n* = 18)	Ponds with no detections (*n* = 8)	Waterway transects (1 occupied; *n* = 26)
Water depth at 1 m (average cm)	16–68 (41)	15–41 (27)	3–80 (21)
Aquatic vegetation cover (average % cover of emergent and submerged vegetation)	6–74 (37)	0.5–74 (26)	1.7–57 (23)
Canopy cover (average %)	0.16–92 (46)	0.74–96 (64)	0–100 (69)

### Detection probability

The most supported model of detection probability included time since sunset and relative humidity as covariates (Appendix 1). From this model, we can infer that detection probability for *P. regilla* call surveys increased with minutes after sunset and decreased as relative humidity increased. This model had three times the support of the constant detection probability model and was therefore used in subsequent occupancy models (Appendix 1).

### Scale of variable influence

For three scale-dependent landscape variables, a single scale of greatest influence on *P. regilla* occupancy was identified based on a model with considerable support in the data (*w*_*i*_ ≥ 0.65) and no models competing (∆AIC_c_ < 2): 250 m for impervious cover, 1500 m for number of ponds, and 2000 m for wetland cover (Table 3). The top-ranked model for road density indicated a scale of 500 m was the best fit; however this model received only moderate support. There were several competing models for tree cover, each with low support (Table 3). For all variables, the covariate value for the scale appearing in the top-ranked model was used in subsequent occupancy analyses. Impervious cover and tree cover had a correlation coefficient of −0.74 (Appendix 2) and had to be modeled separately to test the terrestrial habitat availability hypothesis. For all other hypotheses, model covariates were not collinear (Appendix 2).

**Table 3:** Most supported (∆AICc <2) occupancy models for each scale-dependent landscape variable, using the detection structure *p*(TIME + RH)

Model	AICc	∆AIC_c_	*w*_*i*_	K	-2 L
*Impervious cover*					
*ψ*(250)	79.48	0.0	0.79	5	66.48
*Tree cover*					
*ψ*(500)	90.25	0.0	0.32	5	77.26
*ψ*(1500)	91.16	0.90	0.20	5	78.16
*ψ*(2000)	91.35	1.10	0.19	5	78.36
*ψ*(1000)	91.60	1.35	0.16	5	78.60
*Road density*					
*ψ*(500)	88.21	0.0	0.43	5	75.22
*Number of ponds*					
*ψ*(1500)	85.04	0.0	0.75	5	72.04
*Wetland cover*					
*ψ*(2000)	90.83	0.0	0.65	5	77.82

AICc is a second-order Akaike’s information criterion, for small sample sizes; ∆AICc is the difference in AICc value from top-ranked model; *w*_*i*_ is AICc model weight; K is the number of estimated parameters in the model; −2 L is twice the negative log-likelihood; *ψ* is probability of occupancy

### Occupancy

There was a single competitive model (∆AIC_c_ < 2) for three of the four a priori hypothesis (Table 4). The top-ranked model for the terrestrial habitat hypothesis had full support, and indicated that *P. regilla* occupancy is strongly negatively influenced by the amount of impervious cover within 250 m (IMP250; Table 4). A model that included water depth (DEPTH) as its single occupancy covariate was the top-ranked model for aquatic habitat quality hypothesis; this model, which indicates a slight positive relationship between *P. regilla* occupancy and water depth, received moderate support (Table 4). The most-supported model for the habitat connectivity hypotheses shows that there is some support for the positive influence of the number of ponds within 1500 m (Table 4). There were three competitive models (∆AIC_c_ < 2) representing the non-native predator presence hypothesis; however, the top-ranked model was the constant occupancy (*ψ*(.)) model. There was low support for the competing bullfrog-only (BULLFROG) and fish-only (FISH) models (Table 4) and no support for a combined influence of these predators on *P. regilla* occupancy.

**Table 4:** Most supported (∆AIC_c_ < 2) models for each hypothesis (abbreviated in italics) for *Pseudacris regilla* occupancy

Model	AICc	∆AIC_c_	*w*_*i*_	K	-2 L
*Aquatic habitat quality*					
*ψ*(DEPTH)*p*(TIME + RH)	91.23	0.0	0.43	5	78.22
*Non-native predator presence*					
*ψ*(.)*p*(TIME + RH)	93.49	0.0	0.68	4	83.58
*ψ*(BULLFROG)*p*(TIME + RH)	96.52	0.84	0.15	5	83.52
*ψ*(FISH)*p*(TIME + RH)	96.56	0.88	0.15	5	83.56
*Terrestrial habitat availability*					
*ψ*(IMP250)*p*(TIME + RH)	79.48	0.0	1.0	5	66.48
*Habitat connectivity*					
*ψ*(PONDS1500)*p*(TIME + RH)	85.04	0.0	0.42	5	72.04

*p* is probability of detection; DEPTH is water depth at 1 m from shore; TIME is minutes after sunset; RH is relative humidity; BULLFROG is American bullfrog presence/non-detection; FISH is non-native predatory fish presence/non-detection; IMP250 is impervious cover within 250 m; PONDS1500 is number of ponds within 1500 m of the sample wetland

When the best models representing each hypothesis were compared, the model representing the terrestrial habitat availability hypothesis (IMP250) ranked as the top model with substantial support (Table 5). The only other model with any support was the PONDS1500 model representing the habitat connectivity hypothesis (Table 5). The parametric bootstrap goodness-of-fit test on the top model showed that there was adequate fit (χ^2^ = 3.8, *p* value = 0.61) and no indication of overdispersion (*ĉ* = 0.75). Based on this model, detection probability is estimated as 0.71 and the probability of occupancy is predicted to be 1 for ponds with less than 20% impervious cover and 0 at ponds surrounded by over 35% impervious cover.

**Table 5:** Between-hypothesis occupancy model selection results for *Pseudacris regilla*. Naïve occupancy *ψ*, defined as the estimate of occupancy probability based on the number of positive detections divided by the number of sites, without taking into account occupancy or detection parameters, is shown for comparison. The null model (*ψ*(.)*p*(.)) assumes constant occupancy and detection across sites and call surveys

Model	AICc	∆AIC_c_	*w*_*i*_	K	-2 L	$\hat{\psi}$	SE(*ψ*)	$\hat{p}$
(naïve *ψ* = 0.69)								
*ψ*(IMP250)*p*(TIME + RH)	79.48	0.0	0.94	5	66.48	0.99	0.03	0.71
*ψ*(PONDS1500)*p*(TIME + RH)	85.04	5.56	0.06	5	72.04	0.85	0.12	0.74
*ψ*(DEPTH)*p*(TIME + RH)	91.23	11.75	0.0	5	78.22	0.76	0.11	0.74
*ψ*(.)*p*(.)	93.49	14.01	0.0	4	83.58	0.70	0.09	0.75
*ψ*(BULLFROG)*p*(TIME + RH)	96.52	17.04	0.0	5	83.52	0.67	0.16	0.74
*ψ*(FISH)*p*(TIME + RH)	96.56	17.08	0.0	5	83.56	0.72	0.11	0.74

$\hat{\psi}$ is the estimated proportion of sites occupied (with occupancy covariate set at its mean); SE*(*$\hat{\psi}$) is the standard error of $\hat{\psi};\hat{p}$ is mean detection probability

### Relative variable importance

When the IMP250 model was compared with a global model that combined all covariates from the top models for each hypothesis, and all possible subset models, it remained the top model; however, two other models, which included impervious cover, one or both types of predators, and the number of ponds within 1500 m as covariates, were competing (∆AIC_c_ < 2) (Table 6). The global model received no support. Relative variable importance was calculated from among supported models (*w*_*i*_ > 0). The amount of impervious cover within 250 m of a wetland was identified as the most important factor describing *P. regilla* occurrence with a relative variable weight of 0.91. Bullfrogs, fish, and number of ponds within 1500 m received moderate support, and water depth received the least support (Table 6). Several subset models of the global model would not converge and were removed from the candidate set.

**Table 6:** (A) Model selection results for all subsets of the combined global model using the detection structure *p*(TIME + RH). Note that models without support (*w*_*i*_ = 0) or that did not converge are not shown. Estimates for occupancy(*ψ*) and detection probability (*p*) are calculated with parameters set at their mean. (B) Relative variable importance (*w*_+_) and direction (effect) of model averaged coefficient estimates based on the candidate models for *Pseudacris regilla* occupancy

(A)	Model	AICc	∆AIC_c_	*w*_*i*_	-2 L	K	$\hat{\psi}$	SE(*ψ*)	$\hat{p}$
	*ψ*(IMP250)	79.48	0.0	0.32	66.48	5	0.99	0.026	0.71
	*ψ*(IMP250 + BULLFROG + FISH)	80.49	1.01	0.20	60.26	7	1.0	0.0012	0.71
	*ψ*(IMP250 + PONDS1500 + BULLFROG)	80.49	1.01	0.20	60.26	7	1.0	1.2e^−4^	0.71
	*ψ*(IMP250 + DEPTH)	82.26	2.79	0.08	65.84	6	0.99	0.024	0.71
	*ψ*(IMP250 + FISH)	82.80	3.32	0.06	66.38	6	0.98	0.058	0.71
	*ψ*(PONDS1500 + BULLFROG)	83.43	3.96	0.04	67.02	6	0.93	0.11	0.74
	*ψ*(IMP250 + DEPTH + FISH)	84.40	4.92	0.03	64.18	7	0.86	0.13	0.75
	*ψ*(PONDS1500)	85.04	5.56	0.02	72.04	5	0.85	0.12	0.74
	*ψ*(PONDS1500 + DEPTH)	85.69	6.21	0.01	69.26	6	0.87	0.11	0.74
	*ψ*(PONDS1500 + FISH)	85.81	6.33	0.01	69.38	6	0.91	0.098	0.74
	*ψ*(IMP250 + PONDS1500 + FISH)	86.26	6.78	0.01	66.04	7	0.86	0.14	0.74
	*ψ*(PONDS1500 + BULLFROG + FISH)	86.44	6.96	0.01	66.22	7	0.94	0.086	0.74
(B)	Variable	*w*_+_	Effect						
	IMP250	0.91	–						
	BULLFROG	0.54	–						
	FISH	0.42	–						
	PONDS1500	0.39	+						
	DEPTH	0.23	+						

$\hat{\psi}$ is the estimated proportion of sites occupied (with occupancy covariate set at its mean); SE*(*$\hat{\psi}$) is the standard error of $\hat{\psi};\hat{p}$ is mean detection probability

## Discussion

Our findings lend support to two emerging themes in amphibian conservation in developed landscapes: first, that there is a stronger relationship between wetland occupancy and surrounding terrestrial habitat characteristics than with the characteristics of the wetlands themselves (Lehtinen et al. 1999; Quesnelle et al. 2015; Grand et al. 2017); and second, that amphibian occupancy is driven by multiple factors operating at local and landscape scales (Fischer et al. 2015; Johnson et al. 2016; Marsh et al. 2017). We found that wetland occupancy was driven most strongly by the amount of impervious cover within 250 m of a wetland, underscoring the importance of terrestrial habitat availability for *P. regilla*. We also found a moderate negative association between wetland occupancy and the presence of non-native predators at the local (wetland) scale and a moderate positive association between wetland occupancy and the number of ponds found within 1500 m.

A negative relationship between impervious cover and occupancy has been observed for many aquatic-breeding amphibians (e.g. Knutson et al. 1999; Simon et al. 2009; Marsh et al. 2017) and can be explained by a number of factors. Impervious cover not only displaces essential terrestrial habitat resources, it also limits habitat connectivity by presenting amphibians with barriers to movement and generally high landscape resistance due to risk of desiccation and road mortality. For habitat generalists such as *P. regilla*, which are found in diverse vegetation cover ranging from urban gardens to agricultural fields to open forests, impervious cover can be considered the inverse of suitable terrestrial habitat. Impervious cover can also negatively influence wetland occupancy by increasing surface water flow, leading to extreme water level fluctuations that can strand the eggs of aquatic-breeding amphibians above water (Reinelt et al. 1998; Hayes et al. 2008) causing desiccation and mortality. In addition, stormwater runoff from roads and other impervious surfaces is well known to carry higher concentrations of pollutants (St-Hilaire et al. 2016), which can contaminate receiving wetlands. In this study, only one site was confirmed to be a stormwater treatment pond, while remaining sites were assumed to receive only incidental overland flow. We observed an increase in conductivity with increasing impervious cover (Online Resource 2); thus, another indirect impact of increasing impervious cover is the risk of negative behavioural and physiological effects caused by exposure to increased conductivity, which have been documented for other amphibians (e.g. Sanzo and Hecnar 2006; Karraker et al. 2008; Chambers 2011).

Impervious cover was very strongly negatively associated with *P. regilla* wetland occupancy at the scale of 250 m, and there was no model support for other scales. This suggests that the most important terrestrial habitat for *P. regilla* is found in close proximity to wetlands. It is possible that the 250-m scale was most influential because it is reflective of home range size. To our knowledge, no studies have investigated *P. regilla* home range size directly. One of the earliest studies of *P. regilla* natural history found that most juveniles that had settled post dispersal remained within 200 m of their natal pond, while all juveniles recovered were found within 250 m (Jameson 1956). Other early researchers observed adult *P. regilla* hibernating in small holes in the soil on grassy hills approximately 450 m from a known breeding site (Brattstrom and Warren 1955). Schaub and Larsen (1978) describe the species as relatively sedentary, tending to remain within 10 m of the same pond during the breeding season, but capable of moving up to 400 m. These accounts suggest that *P. regilla* home range is 250–500 m around a wetland in undisturbed habitats, and it is possible that urban and agricultural development restricts home range size to the lower limit of this range. However, mark-recapture and telemetry studies, particularly those which quantify movement across scales and relate this to individual or population parameters, are needed to understand the optimal home range size for *P. regilla* in a developed landscape (Bailey and Muths 2019).

Multiscale models that included non-native predator covariates alongside landscape-scale covariates were competitive with the most supported model (Table 6a). The presence of American bullfrogs and non-native fish had a moderately negative and slightly negative effect on *P. regilla* wetland occupancy, respectively (Table 6b). A negative effect of non-native predators was expected, as there is ample evidence that bullfrogs displace, compete with, and predate on, native amphibians (Kiesecker and Blaustein 1998; Kiesecker et al. 2001; Pearl et al. 2004; Rowe and Garcia 2014), and that non-native fish predate on, and facilitate bullfrog predation of, native amphibians (Pearl et al. 2005; Rowe and Garcia 2014; Guderyahn et al. 2016). The moderate to weak negative associations with *P. regilla* occupancy are consistent with other reports, and lend support to the theory that bullfrogs and non-native fish are negatively impacting amphibians in western North America without completely excluding them (Adams 1999; Govindarajulu 2004; Pearl et al. 2005; Holzer 2014). Nevertheless, the negative impacts of non-native predators can be mitigated with restoration and creation of wetlands that have hydroperiods sufficient for metamorphosis in native species, but which do not support the permanent water requirements of American bullfrogs and fish (Snodgrass et al. 2000; Govindarajulu 2004; Pearl et al. 2005).

As with the non-native predators hypothesis, models focused exclusively on habitat connectivity were not competitive (Table 5), but a habitat connectivity variable appeared in competitive models when combined with other covariates (Table 6a). Specifically, the number of ponds within 1500 m was identified as having a moderate, positive influence on *P. regilla* occupancy (Table 6b). The positive association between *P. regilla* occupancy and the number of ponds within 1500 m suggests that *P. regilla* is reliant, to some degree, on occasional long-distance dispersal events to maintain local populations, and that the likelihood of occurrence increases as the number of source and “stepping stone” ponds within a maximum dispersal distance increases (Watts et al. 2015). While *P. regilla* appear to show strong site fidelity and do not frequently make long-distance movements, they are capable of moving at least 1900 m in search of suitable habitat post displacement (Smith and Green 2005). Model support for the number of ponds within 1500 m suggests that this may be the scale at which metapopulation dynamics play out for *P. regilla* in a highly fragmented urban or rural landscape; however, focused research on *P. regilla* dispersal and local and regional population dynamics is needed to test this hypothesis (Smith and Green 2005).

The remaining variables we considered had little influence on *P. regilla* occupancy. For aquatic habitat variables, is possible that our measurements were within tolerable ranges. Alternatively, effects might have been detected if we had collected multiple measurements throughout the breeding season and used an average. Among the remaining landscape-scale variables, we were most surprised at the lack of influence of road density on occupancy. The negative impact of roads on amphibian population persistence is well documented (Beebee 2013). Roads contribute significantly to amphibian mortality via vehicle collisions and exposure to pollutants (e.g., Lefcort et al. 1997; Garrah et al. 2015). Furthermore, high levels of ambient traffic noise have been shown to significantly mask the breeding calls of *P. regilla*, who are not able to alter the volume, duration or timing of their calls in response to levels of ambient noise (Nelson et al. 2017). Diminished communication could impede breeding activities such as breeding site orientation and mate selection, with long term consequences for population persistence (Nelson et al. 2017). These documented impacts of roads may explain the absence of *P. regilla* from ditch sites, the majority of which were located adjacent to roads.

### Caveats

The main caveats for this study are that (1) the presence of chorusing males is not evidence of successful breeding or survival of offspring; therefore, further research of other life stages is needed; (2) the identified species-habitat relationships are correlational rather than causal, although known ecological mechanisms explain the observed relationships; (3) there may be other habitat characteristics that are influential that were not considered in this study; and (4) an ability to detect effects and estimate model parameters with a high level of precision are limited by a relatively small sample size. Nevertheless, consistency between the most-supported models and relative variable weights allow some confidence in the quality of the results of this model framework.

### Management implications

As urban and agricultural development expands at the expense of naturally-occurring ecosystems, the conservation and enhancement of biodiversity *within* the developed landscape will take on even more importance. Species such as *P. regilla* are valuable flagships for achieving biodiversity conservation goals in developed areas, and should be included in wildlife management programs. Our study identifies potential drivers of *P. regilla* occupancy, and the scales of influence of these drivers, to provide initial guidance for land managers. First, thoughtful and innovative approaches to development that limit impervious surface cover to 20% within 250 m of potential aquatic habitat should improve the likelihood of *P. regilla* occurrence, particularly if vegetation is maintained on the remaining landscape to provide sufficient complimentary terrestrial habitat. Second, when selecting sites for breeding pond restoration or creation, managers could consider placing ponds where pond density at a scale of 1500 m is maximized. Dispersal can be further supported by maintaining structural connectivity and enhancing matrix permeability, including protection of movement corridors (Churko et al. 2020) and strategic placement of road mitigation structures (Hamer et al. 2015). Third, populations of *P. regilla* that co-occur with American bullfrogs and/or non-native fish should be monitored to detect long-term impacts. Where possible, created and restored ponds should be designed to have a temporary hydroperiod to exclude bullfrogs and fish. Finally, pond restoration or creation designs should consider a wide depth gradient within the littoral zone, as we detected a weak positive relationship with water depth that is supported by the literature (Dupré and Petranka 1985; Kiesecker and Blaustein 1998; Guderyahn et al. 2016).

### Conclusion

This study demonstrates that *P. regilla* can still be found chorusing in the second-most populous region of British Columbia, in urban and rural areas, in relict natural ponds as well as in the novel and designed ecosystems that are becoming increasingly common (e.g. Monello and Wright 1999; Holzer and Lawler 2015; Higgs 2017). However, our findings suggest that the likelihood of *P. regilla* occupancy of a given wetland will decrease as surrounding development intensifies unless impervious cover is heavily restricted and nearby ponds are protected. A proactive approach to *P. regilla* conservation would be to identify and protect existing habitat and create additional habitat using the suitability criteria identified here as a general guide and point of departure for further research (Sterrett et al. 2019). In turn, *P. regilla* can be used as a flagship species to further the restoration and conservation of regional biodiversity.

### Electronic supplementary material

ESM 1 (PDF 11 kb)

ESM 2 (PDF 292 kb)

## Appendix 1

Model selection results for detection probability. ΔAIC <2 indicates substantial support in the data. Models including all other sampling covariates had less support than the null model *ψ*(.)*p*(.) and are not shown here. AICc is a second-order Akaike’s information criterion for small sample sizes; ∆AICc is difference in AICc value from the top-ranked model; *w*_*i*_ is AICc model weight; K is number of estimated parameters in the model; −2 L is twice the negative log-likelihood; $\hat{p}$ is estimated detection probability. Covariate abbreviations: TIME is minutes since sunset; RH is relative humidity; DIST is anthropogenic noise disturbance; BULLFROG is American bullfrog presence/non-detection.

**Table:**

Model	AIC_c_	∆AIC_c_	*w*_*i*_	K	-2 L	$\hat{p}$
*ψ*(.)*p*(TIME + RH)	93.49	0.0	0.18	4	83.58	0.74
*ψ*(.)*p*(DIST + BULLFROG)	93.60	0.11	0.17	4	83.70	0.68
*ψ*(.)*p*(DIST)	93.83	0.34	0.15	3	86.74	0.68
*ψ*(.)*p*(RH)	94.71	1.22	0.10	3	87.62	0.68
*ψ*(.)*p*(TIME + DIST)	95.30	1.81	0.07	4	85.40	0.75
*ψ*(.)*p*(BULLFROG)	95.33	1.84	0.07	3	88.24	0.74
*ψ*(.)*p*(TIME + BULLFROG)	95.43	1.94	0.07	4	85.52	0.74
*ψ*(.)*p*(RH + BULLFROG)	95.60	2.10	0.06	4	85.70	0.75
*ψ*(.)*p*(TIME)	95.67	2.17	0.06	3	88.58	0.75
*ψ*(.)*p*(.)	95.68	2.19	0.06	2	91.16	0.75

## Appendix 2

Correlation coefficients for modeled covariates. Bold font indicates that the covariate pair is highly correlated and should not be included in the same model. Covariate abbreviations: AREA is aquatic footprint (m^2^); CANOPY is percent canopy cover over the shoreline; AQVEG is percent cover of emergent/submerged vegetation within 1 m of shore; DEPTH is water depth at 1 m from shore (cm); BULLFROG is American bullfrog presence/non-detection; FISH is non-native predatory fish presence/non-detection; NEAR is distance to nearest neighbour pond (m); RD500 is road density (m/m^2^) within 500 m; PONDS1500 is a count of the number of ponds within 1500 m; WET2000 is percent cover of wetlands within 2000 m; TREE500 is percent tree cover within 500 m; IMP250 is percent impervious cover within 250 m; TREE2000 is percent tree cover within 2000 m.

**Table:**

	CANOPY	AQVEG	DEPTH	BULLFROG	FISH	NEAR	RD500	PONDS1500	WET2000	TREE500	IMP250	TREE2000
AREA	─0.15	0.16	─0.24	0.19	0.46	─0.16	─0.29	0.24	0.02	0.21	─0.34	0.33
CANOPY	–	─0.41	─0.05	─0.14	0.13	0.09	0.16	0.05	0.22	0.21	0.00	0.04
AQVEG		–	0.10	0.31	─0.11	─0.14	─0.21	0.16	0.08	─0.02	─0.10	0.07
DEPTH			–	─0.04	0.16	0.04	─0.36	0.33	0.41	0.10	─0.21	0.30
BULLFROG				–	0.19	─0.24	─0.54	0.44	0.26	0.34	─0.45	**0.56**
FISH					–	0.10	─0.41	0.21	0.16	0.29	─0.17	0.35
NEAR						–	0.29	─0.44	─0.28	─0.07	0.24	−0.33
RD500							–	**─0.70**	─0.45	─0.54	**0.80**	**─0.60**
PONDS1500								–	0.53	**0.59**	**─0.69**	**0.78**
WET2000									–	0.45	─0.40	0.48
TREE500										–	**─0.74**	**0.68**
IMP250											–	**─0.58**

## References

[CR1] Adams MJ (1999). Correlated factors in amphibian decline: exotic species and habitat change in Western Washington. J Wildl Manag.

[CR2] Akaike H, Petrov BN, Csaki F (1973). Information theory as an extension of the maximum likelihood principle. Second international symposium on information theory.

[CR3] Altmoos M, Henle K (2010) Relevance of multiple spatial scales in habitat models: a case study with amphibians and grasshoppers. Acta Oecol 36:548–560. 10.1016/j.actao.2010.08.001

[CR4] Bailey LL, Muths E (2019). Integrating amphibian movement studies across scales better informs conservation decisions. Biol Conserv.

[CR5] Baldwin RF, DeMaynadier PG (2009). Assessing threats to pool-breeding amphibian habitat in an urbanizing landscape. Biol Conserv.

[CR6] Beebee TJC (2013). Effects of road mortality and mitigation measures on amphibian populations. Conserv Biol.

[CR7] Bishop MR, Drewes RC, Vredenburg VT (2014). Food web linkages demonstrate importance of terrestrial prey for the threatened California red-legged frog. J Herpetol.

[CR8] Bjorkman AD, Vellend M (2010) Defining historical baselines for conservation: ecological changes since European settlement on Vancouver Island, Canada. Conserv Biol 24:1559–1568. 10.1111/j.1523-1739.2010.01550.x 10.1111/j.1523-1739.2010.01550.x 20586787

[CR9] Blaustein AR, Romansic JM, Kiesecker JM (2003). Ultraviolet radiation, toxic chemicals and amphibian population declines. Divers Distrib.

[CR10] Boissinot A, Besnard A, Lourdais O (2019). Amphibian diversity in farmlands: combined influences of breeding-site and landscape attributes in western France. Agric Ecosyst Environ.

[CR11] Brand AB, Snodgrass JW (2010) Value of artificial habitats for amphibian reproduction in altered landscapes. Conserv Biol 24:295–301. 10.1111/j.1523-1739.2009.01301.x 10.1111/j.1523-1739.2009.01301.x 19681986

[CR12] Brattstrom BH, Warren JW (1955). Observations on the ecology and behavior of the Pacific Treefrog, Hyla regilla. Copeia.

[CR13] Burnham KP, Anderson DR (2002). Model selection and multimodel inference: a practical information-theoretic approach.

[CR14] Chambers DL (2011). Increased conductivity affects corticosterone levels and prey consumption in larval amphibians. J Herpetol.

[CR15] Churko G, Kienast F, Bolliger J (2020). A multispecies assessment to identify the functional connectivity of amphibians in a human-dominated landscape. ISPRS Int J Geo-Information.

[CR16] Cox RK, Cullington J (2009) Wetland ways: interim guidelines for wetland protection and conservation in British Columbia. Wetland Stewardship Partnership

[CR17] Cushman SA (2006). Effects of habitat loss and fragmentation on amphibians: a review and prospectus. Biol Conserv.

[CR18] Dorcas ME, Price SJ, Walls SC, Barichivich WJ, Dodd CK (2010). Auditory monitoring of anuran populations. Amphibian ecology and conservation: a handbook of techniques.

[CR19] Dupré RK, Petranka JW (1985). Ontogeny of temperature selection in larval amphibians. Copeia.

[CR20] Egea-Serrano A, Relyea RA, Tejedo M, Torralva M (2012). Understanding of the impact of chemicals on amphibians: a meta-analytic review. Ecol Evol.

[CR21] ESRI (2017). ArcMap.

[CR22] Fischer K, Becker M, Becker BA, Bensch J, Böckers A, Burmeister M, Dombrowski J, Donke E, Ermisch R, Fritze M, Fritzsch A, Hübler N, Ide M, Klockmann M, Mielke M, Pfender D, Schiffler M, Schrödter M, Sund L, Viertel C, Weise E, Werner M, Winter M (2015). Determinants of tree frog calling ponds in a human-transformed landscape. Ecol Res.

[CR23] Fiske I, Chandler R (2011). Unmarked: an R package for fitting hierarchical models of wildlife occurrence and abundance. J Stat Softw.

[CR24] Garcia-Gonzalez C, Garcia-Vazquez E (2012). Urban ponds, neglected Noah’s ark for amphibians. J Herpetol.

[CR25] Garrah E, Danby RK, Eberhardt E, Cunnington GM, Mitchell S (2015). Hot spots and hot times: wildlife road mortality in a regional conservation corridor. Environ Manag.

[CR26] GOERT (Garry Oak Ecosystems Recovery Team) (2003) Garry oak ecosystems recovery team research colloquium 2003. Victoria, BC, pp 1–22

[CR27] Goldberg CS, Waits LP (2009). Using habitat models to determine conservation priorities for pond-breeding amphibians in a privately-owned landscape of northern Idaho, USA. Biol Conserv.

[CR28] Govindarajulu P (2004) Introduced bullfrogs (*Rana catesbeiana*) in British Columbia: impacts on native Pacific Treefrogs (*Hyla regilla*) and red-legged frogs (*Rana aurora*). PhD Dissertation, University of Victoria

[CR29] Grand LA, Hayes MP, Vogt KA, Vogt DJ, Yarnold PR, Richter KO, Anderson CD, Ostergaard EC, Wilhelm JO (2017). Identification of habitat controls on northern red-legged frog populations: implications for habitat conservation on an urbanizing landscape in the Pacific northwest. Ecol Process.

[CR30] Grant EHC, Miller DAW, Schmidt BR, Adams MJ, Amburgey SM, Chambert T, Cruickshank SS, Fisher RN, Green DM, Hossack BR, Johnson PTJ, Joseph MB, Rittenhouse TAG, Ryan ME, Waddle JH, Walls SC, Bailey LL, Fellers GM, Gorman TA, Ray AM, Pilliod DS, Price SJ, Saenz D, Sadinski W, Muths E (2016). Quantitative evidence for the effects of multiple drivers on continental-scale amphibian declines. Sci Rep.

[CR31] Guderyahn LB, Smithers AP, Mims MC (2016). Assessing habitat requirements of pond-breeding amphibians in a highly urbanized landscape: implications for management. Urban Ecosyst.

[CR32] Hamer AJ, McDonnell MJ (2008). Amphibian ecology and conservation in the urbanising world: a review. Biol Conserv.

[CR33] Hamer AJ, Langton TES, Lesbarrères D, van der Ree R, Smith DJ, Grilo C, Smith D (2015). Making a safe leap forward: mitigating road impacts on amphibians. Handbook of road ecology.

[CR34] Hayes MP, Quinn T, Richter KO et al (2008) Maintaining lentic-breeding amphibians in urbanizing landscapes: the case study of the northern red-legged frog (Rana aurora). In: Mitchell JC, Jung Brown RE (eds) Urban Herpetology. Society for the Study of Amphibians and Reptiles, pp 133–149

[CR35] Higgs E (2017). Novel and designed ecosystems. Restor Ecol.

[CR36] Hillman SS, Drewes RC, Hedrick MS, Hancock TV (2014). Physiological vagility: correlations with dispersal and population genetic structure of amphibians. Physiol Biochem Zool Ecol Evol Approaches.

[CR37] Hof C, Araújo MB, Jetz W, Rahbek C (2011). Additive threats from pathogens, climate and land-use change for global amphibian diversity. Nature.

[CR38] Holzer KA (2014). Amphibian use of constructed and remnant wetlands in an urban landscape. Urban Ecosyst.

[CR39] Holzer KA, Lawler SP (2015). Introduced reed canary grass attracts and supports a common native amphibian. J Wildl Manag.

[CR40] Hossack BR (2017). Amphibian dynamics in constructed ponds on a wildlife refuge: developing expected responses to hydrological restoration. Hydrobiologia.

[CR41] Houlahan JE, Findlay CS, Schmidt BR, Meyer AH, Kuzmin SL (2000). Quantitative evidence for global amphibian population declines. Nature.

[CR42] Hurvich CM, Tsai C-L (1989). Regression and time series model selection in small samples. Biometrika.

[CR43] IUCN (2020) The IUCN Red List of Threatened Species. Version 2020-1. https://www.iucnredlist.org. Accessed 15 May 2020

[CR44] Jameson DL (1956). Growth, dispersal and survival of the Pacific tree frog. Copeia.

[CR45] Johnson PTJ, Hoverman JT, McKenzie VJ (2013). Urbanization and wetland communities: applying metacommunity theory to understand the local and landscape effects. J Appl Ecol.

[CR46] Johnson BA, Barrett K, Homyack JA, Baldwin RF (2016). Anuran occupancy and breeding site use of aquatic systems in a managed pine landscape. For Ecol Manag.

[CR47] Karraker NE, Gibbs JP, Vonesh JR (2008). Impacts of road deicing salt on the demography of vernal pool-breeding amphibians. Ecol Appl.

[CR48] Kerby JL, Richards-Hrdlicka KL, Storfer A, Skelly DK (2010). An examination of amphibian sensitivity to environmental contaminants: are amphibians poor canaries?. Ecol Lett.

[CR49] Kiesecker JM, Blaustein AR (1998). Effects of introduced bullfrogs and smallmouth bass on microhabitat use, growth, and survival of native red-legged frogs (Rana aurora). Conserv Biol.

[CR50] Kiesecker JM, Blaustein AR, Miller CL (2001). Potential mechanisms underlying the displacement of native red-legged frogs by introduced bullfrogs. Ecology.

[CR51] Knutson MG, Sauer JR, Olsen DA, Mossman MJ, Hemesath LM, Lannoo MJ (1999). Effects of landscape composition and wetland fragementation on frog and toad abundance and species richness in Iowa and Wisconsin, United States of America. Conserv Biol.

[CR52] Lefcort H, Hancock KA, Maur KM, Rostal DC (1997). The effects of used motor oil, silt, and the water mold Saprolegnia parasitica on the growth and survival of mole salamanders (genus Ambystoma). Arch Environ Contam Toxicol.

[CR53] Lehtinen RM, Galatowitsch SM, Tester JR (1999). Consequences of habitat loss and fragmentation for wetland amphibian assemblages. Wetlands.

[CR54] MacKenzie DI, Bailey LL (2004). Assessing the fit of site-occupancy models. J Agric Biol Environ Stat.

[CR55] MacKenzie DI, Royle JA (2005). Designing occupancy studies: general advice and allocating survey effort. J Appl Ecol.

[CR56] MacKenzie DI, Nichols JD, Lachman GB (2002). Estimating site occupancy rates when detection probabilities are less than one. Ecology.

[CR57] MacKenzie DI, Nichols JD, Royle JA (2006). Occupancy estimation and modeling: inferring patterns and dynamics of species occurrence.

[CR58] Marco A, Quilchano C, Blaustein AR (1999). Sensitivity to nitrate and nitrite in pond-breeding amphibians from the Pacific northwest, USA. Environ Toxicol Chem.

[CR59] Marsh DM, Trenham PC (2001). Metapopulation dynamics and amphibian conservation. Conserv Biol.

[CR60] Marsh DM, Cosentino BJ, Jones KS, Apodaca JJ, Beard KH, Bell JM, Bozarth C, Carper D, Charbonnier JF, Dantas A, Forys EA, Foster M, General J, Genet KS, Hanneken M, Hess KR, Hill SA, Iqbal F, Karraker NE, Kilpatrick ES, Langen TA, Langford J, Lauer K, McCarthy AJ, Neale J, Patel S, Patton A, Southwick C, Stearrett N, Steijn N, Tasleem M, Taylor JM, Vonesh JR (2017). Effects of roads and land use on frog distributions across spatial scales and regions in the eastern and Central United States. Divers Distrib.

[CR61] Matsuda BM, Green DM, Gregory PT (2006). Amphibians and reptiles of British Columbia.

[CR62] Miguet P, Jackson HB, Jackson ND, Martin AE, Fahrig L (2016). What determines the spatial extent of landscape effects on species?. Landsc Ecol.

[CR63] Monello RJ, Wright RG (1999). Amphibian habitat preferences among artificial ponds in the Palouse region of northern Idaho. J Herpetol.

[CR64] Nelson DV, Klinck H, Carbaugh-Rutland A, Mathis CL, Morzillo AT, Garcia TS (2017). Calling at the highway: the spatiotemporal constraint of road noise on Pacific chorus frog communication. Ecol Evol.

[CR65] NOAA (National Oceanic and Atmospheric Administration) (2013) Sampling Design Tool for ArcGIS. National Centers for Coastal Ocean Science, Silver Spring, MD

[CR66] Nori J, Villalobos F, Loyola R (2018). Global priority areas for amphibian research. J Biogeogr.

[CR67] Ovaska K, Davis TM, Flamarique IN (1997). Hatching success and larval survival of the frogs Hyla regilla and Rana aurora under ambient and artificially enhanced solar ultraviolet radiation. Can J Zool Rev Can Zool.

[CR68] Pearl CA, Adams MJ, Bury RB, McCreary B (2004). Asymmetrical effects of introduced bullfrogs (Rana catesbeiana) on native Ranid frogs in Oregon. Copeia.

[CR69] Pearl CA, Adams MJ, Leuthold N, Bury RB (2005). Amphibian occurrence and aquatic invaders in a changing landscape: implications for wetland mitigation in the Willamette Valley, Oregon, USA. Wetlands.

[CR70] Petranka JW, Smith CK, Scott AF (2004). Identifying the minimal demographic unit for monitoring pond-breeding amphibians. Ecol Appl.

[CR71] Quesnelle PE, Lindsay KE, Fahrig L (2015). Relative effects of landscape-scale wetland amount and landscape matrix quality on wetland vertebrates: a meta-analysis. Ecol Appl.

[CR72] R Core Team (2017) R: A language and environment for statistical computing. R Foundation for Statistical Computing, Vienna, Austria

[CR73] Reinelt L, Horner R, Azous A (1998). Impacts of urbanization on palustrine (depressional freshwater) wetlands—research and management in the Puget Sound region. Urban Ecosyst.

[CR74] Riley SPD, Busteed GT, Kats LB et al (2005) Effects of urbanization on the distribution and abundance of amphibians and invasive species in Southern California streams. Conserv Biol 19:1894–1907. https://doi.org/10.1111/j

[CR75] Rorabaugh JC, Lannoo MJ, Lannoo MJ (2005). Pseudacris regilla (Baird and Girard, 1852[b]) Pacific Treefrog. Amphibian declines: the conservation status of United States species.

[CR76] Rowe JC, Garcia TS (2014). Impacts of wetland restoration efforts on an amphibian assemblage in a multi-invader community. Wetlands.

[CR77] Saarikivi J, Knopp T, Granroth A, Merilä J (2013). The role of golf courses in maintaining genetic connectivity between common frog (Rana temporaria) populations in an urban setting. Conserv Genet.

[CR78] Sanzo D, Hecnar SJ (2006). Effects of road de-icing salt (NaCl) on larval wood frogs (Rana sylvatica). Environ Pollut.

[CR79] Schaub DL, Larsen JH (1978). The reproductive ecology of the Pacific Treefrog (Hyla regilla). Herpetologica.

[CR80] Scheffers BR, Paszkowski CA (2012). The effects of urbanization on north American amphibian species: identifying new directions for urban conservation. Urban Ecosyst.

[CR81] Semlitsch RD (2002) Critical elements for biologically based recovery plans of aquatic-breeding amphibians. Conserv Biol 16:619–629. 10.1046/j.1523-1739.2002.00512.x

[CR82] Shaffer HB, Alford RA, Woodward BD, Heyer R, Donnelly MA, Foster M, Mcdiarmid R (1994). Quantitative sampling of amphibian larvae. Measuring and monitoring biological diversity: standard methods for amphibians.

[CR83] Simon JA, Snodgrass JW, Casey RE, Sparling DW (2009). Spatial correlates of amphibian use of constructed wetlands in an urban landscape. Landsc Ecol.

[CR84] Skelly DK, Richardson JL (2010) Larval sampling. In: Dodd CK (ed) Amphibian ecology and conservation: a handbook of techniques. Oxford University Press, pp 55–70

[CR85] Smith MA, Green DM (2005). Dispersal and the metapopulation paradigm in amphibian ecology and conservation: are all amphibian populations metapopulations ?. Ecography (Cop).

[CR86] Snodgrass JW, Komoroski MJ, Bryan LA, Burger J (2000). Relationships among isolated wetland size, hydroperiod, and amphibian species richness: implications for wetland regulations. Conserv Biol.

[CR87] Sparling DW, Fellers GM (2009). Toxicity of two insecticides to California, USA, anurans and its relevance to declining amphibian populations. Environ Toxicol Chem.

[CR88] Sterrett SC, Katz RA, Brand AB, Fields WR, Dietrich AE, Hocking DJ, Foreman TM, Wiewel ANM, Campbell Grant EH (2019). Proactive management of amphibians: challenges and opportunities. Biol Conserv.

[CR89] St-Hilaire A, Duchesne S, Rousseau AN (2016). Floods and water quality in Canada: a review of the interactions with urbanization, agriculture and forestry. Can Water Resour J.

[CR90] Stuart SN, Chanson JS, Cox NA (2004). Status and trends of amphibian declines and extinctions worldwide. Science (80- ).

[CR91] U.S. EPA (2002). Methods for evaluating wetland condition: using amphibians in bioassessments of wetlands.

[CR92] Urban Forest Stewardship Initiative (2008) Forest & land cover mapping. https://www.hat.bc.ca/our-blog/urban-forest-stewardship-initiative. Accessed 18 May 2018

[CR93] Watts AG, Schlichting PE, Billerman SM, Jesmer BR, Micheletti S, Fortin MJ, Funk WC, Hapeman P, Muths E, Murphy MA (2015) How spatio-temporal habitat connectivity affects amphibian genetic structure. Front Genet 6:1–13. 10.3389/fgene.2015.00275 10.3389/fgene.2015.00275 PMC4561841 26442094

[CR94] Weir LA, Mossman MJ, Lannoo MJ (2005). North American amphibian monitoring program (NAAMP). Amphibian declines: the conservation status of United States species.

[CR95] Wells KD (2010). The ecology and behavior of amphibians.

[CR96] Zuur AF, Ieno EN, Elphick CS (2010). A protocol for data exploration to avoid common statistical problems. Methods Ecol Evol.

