# Supplementary material for: Multiscale determinants of Pacific chorus frog occurrence in a developed landscape
Source: Urban Ecosyst. 2020 Oct 8;24(3):587–600. doi: 10.1007/s11252-020-01057-4 (PMC8550069; doi:10.1007/s11252-020-01057-4)
Supplement: Supplementary file 1 — (PDF 11 kb) [file 11252_2020_1057_MOESM1_ESM.pdf]

Ordinal sampling covariates and associated code descriptions. Wind speed, cloud cover, and anthropogenic disturbance codes adapted from Weir and Mossman (2005).

**Variable / code**	**Description**
**Wind Speed**	
0	Calm (<1.6 km/hr)
1	Slight (1.6-4.8 km/hr)
2	Light breeze (4.9-11.3 km/hr)
3	Gentle breeze (11.4-19.3 km/hr)
**Cloud Cover**	
0	Clear or few clouds
1	Partly cloudy (scattered or variable)
2	Overcast
**Precipitation**	
0	None
1	Drizzle
2	Steady light rain
3	Showers affecting hearing ability (pause or discontinue surveys)
**Moon brightness**	
0	No moon or overcast
1	Quarter moon unobstructed or later phase mostly obstructed
2	Half-moon unobstructed or later phase partly obstructed
3	Three-quarter moon unobstructed or later phase slightly obstructed
4	Full moon, unobstructed
**Anthropogenic disturbance**	
0	No appreciable effect
1	Slightly affecting sampling (distant traffic or a single car passing)
2	Moderately affecting sampling (nearby traffic or 2-5 cars passing)
3	Seriously affecting sampling
