# Supplementary material for: Multiscale determinants of Pacific chorus frog occurrence in a developed landscape
Source: Urban Ecosyst. 2020 Oct 8;24(3):587–600. doi: 10.1007/s11252-020-01057-4 (PMC8550069; doi:10.1007/s11252-020-01057-4)

The positive relationship between water conductivity (µS) and impervious surface cover within 250 m of study ponds

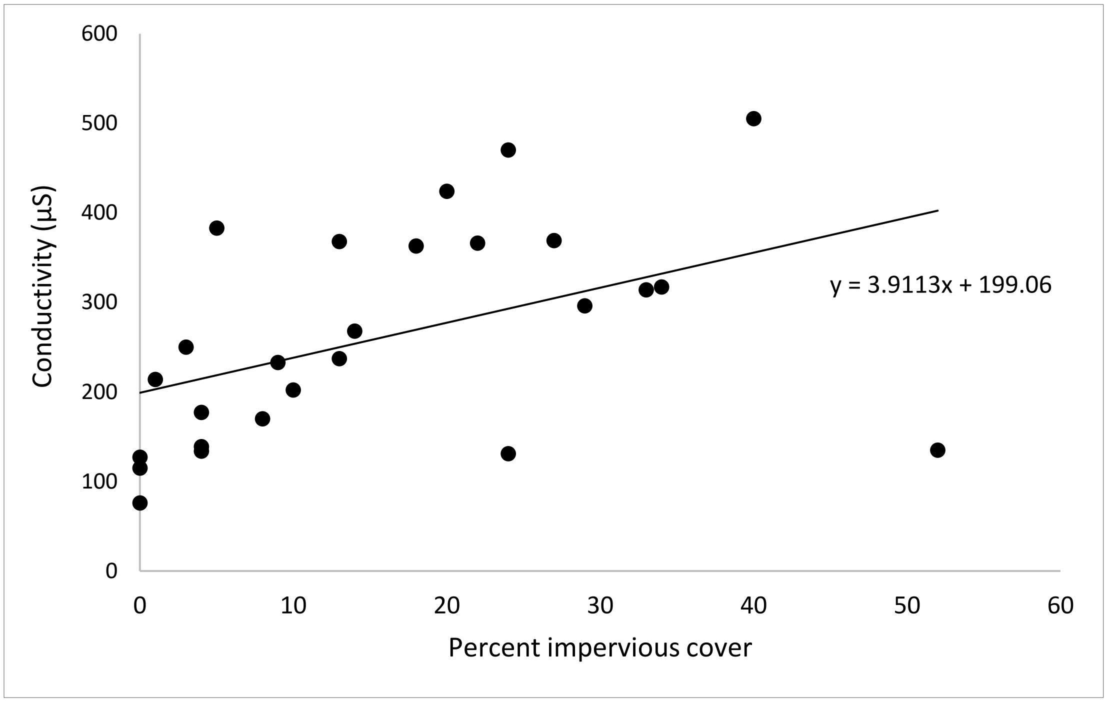

Supplement: Supplementary file 2 — (PDF 292 kb) [file 11252_2020_1057_MOESM2_ESM.pdf]
